# Medicinal potential of *Morella serata* (Lam.) Killick (Myricaceae) root extracts: biological and pharmacological activities

**DOI:** 10.1186/1472-6882-13-163

**Published:** 2013-07-08

**Authors:** Anofi Omotayo Tom Ashafa

**Affiliations:** 1 Phytomedicine and Phytopharmacological Research Group, Department of Plant Sciences, University of the Free State, Qwaqwa Campus, Phuthaditjhaba 9866, South Africa

**Keywords:** *Morella serata* (Monna motsho), Biological and pharmacological activities, Antitumor, Infectious diseases

## Abstract

**Background:**

*Morella serata* is a South African medicinal plant used in the treatment of microbial infections and to enhance male sexual performance. There is dearth of information in scientific literature on its efficacy and safety.

**Methods:**

In the present study, the root extracts were investigated for the phytochemicals that may be present the antibacterial, anticandida activity using 96 wells microtitre plate method and cytotoxicity using brine shrimp (*Artemia salina*) lethality assay.

**Results:**

The qualitative phytochemical screening revealed the presence of tannins, saponins, flavonoids, terpenoids and steroids. All the extracts including water inhibited both Gram positive and Gram negative bacteria strains at minimum inhibitory concentrations (MIC) ranging from 0.09 – 6.25 mgmL^-1^. The best activity was observed in the acetone extract inhibiting all the bacteria tested at MIC range of 0.09 – 0.78 mgmL^-1^ except *Shigella flexneri* KZN that was inhibited at 1.56 mgmL^-1^. Similarly, all the extracts suppressed the growth of all Candida species and *Trichophyton mucoides* at MIC ranging from 0.13 – 3.13 mgmL^-1^. The cytotoxicity assay revealed potent cytotoxic potential of *M. serata* methanol and ethanol root extracts by displaying LC_50_ of 0.26 and 0.18 μgmL^-1^ respectively.

**Conclusion:**

The results obtained from the present study indicated broad spectrum antimicrobial activity and justifies the use of the plant in the treatment of infectious diseases. Also the species could be a good natural source of antitumor compounds considering its lethality against brine shrimp nauplii.

## Background

Plants are used medicinally in different countries of the world and are good source of many potent and powerful drugs [1]. In Africa, the use of plant derived remedies in traditional health care practices is common and widespread [2], even before the introduction of synthetic antibiotics and other modern drugs [3]. Today, about 80% of the World’s population still depends on the traditional medicine practices for the management of various diseases [3,4].

Resistance of microbes (bacteria, fungi and viruses) to available antimicrobial agents is a major global public health problem [5]. Over the last few decades, the incidence of opportunistic infections in patients treated with immunosuppressive drugs or intensive chemotherapy is increasing at an alarming rate [6]. Infective diseases accounts for approximately one half of all the deaths in most developing countries of the world. Several new strategies to control such infections caused by microbes have been considered in recent years. Such strategies include the use antibiotic combination, the development of new members of existing antibiotic classes and the introduction of novel agents [6,7]. The problems of microbial drug resistance, an increase of opportunistic infections and the toxicity effect of continued use of several antimicrobial agents have necessitated a search for new antimicrobial drugs from other sources including plants which are the good raw materials for novel antimicrobial chemotherapeutic agents [8,9].

Although there is a growing interest in correlating the phytochemical constituents of a medicinal plant with its pharmacological activity [10,11], the toxicological effects of most of these crude extracts are often overlooked based on the facts that plant medicines have better compatibilities with the human body and produce fewer side effects [3]. However, to forestall adverse effects, sometimes life-threatning, allegedly arising from herbal remedies consumption, cytotoxicity testing, an integral component of the biological evaluation of pharmacological materials and an essential part of standard procedures becomes necessary [3].

*M. serata* (Lam.) Killick formerly called *Myrica serata* Lam. is also known as Lance-leaved waxberry (English), Smalblaarwasbessie (Afrikaans), Umqhwetayo (Xhosa), Umlulama, uMakhutula (Zulu), Uleti (Swaziland) and Monnamotsho (Sotho) is a densely leafed, multi-stem shrub growing up to 2 m tall in colonies of damp grassland. The leaves are narrow, toothed and about 80 mm long tapering at both base and apex [12]. The species is indigenous South and southern Africa, and widespread from the Western Cape, northwards through most southern African countries extending into tropical Africa [13]. In the traditional medicine practice of the Xhosa, the plant is used to treat chest related problems such as asthma, coughing and shortness of breath. A decoction of the root tastes like ginger and causes irritation of the throat, therefore, the name umqhwetayo (the itchy one) [13]. Among the Basotho of the eastern Free State Province, the decoction of the root is used for painful menstruation, cold, coughs and headaches [14], and to enhance men sexual performance, hence the name Monna-motsho (black man). It is also used in the management of sugar related disorder and as laxative to treat constipation. In Zimbabwe and Swaziland, the plant is cut for fuel and the stem bark and root are used to treat headache and as an insurance against bad luck. Till date, little or no information is available in the scientific literature validating the use of *M. serata* in the South African folkloric medicine. Therefore the present study was aimed at investigating the phytoconstituents in order to correlate the folkloric claims with the bioactive compounds present in the plant. Also to evaluate the antibiotic bioprospecting potential of the species and finally to investigate the cytotoxicity potential of the plant on brine shrimp (*Artemia salina*) larvae. Since toxicological evaluation of plant extracts seeks to determine its possible collateral effects to ensure the safety of its use, brine shrimp larvae being sensitive to toxic substances are commonly used for toxicity assays in pharmacology [3,15]. This paper present for the first time, the qualitative phytochemical screening, antibacterial, anticandida and cytotoxicity of *M. serata* root extracts.

## Methods

### Plant collection

The plant material was purchased from traditional herbal vendor at Qwaqwa (Sesting), Free State Province in December 2012, The species was authenticated by Dr. Erwin Sieben of the department of Plant Sciences, University of the Free State, Qwaqwa Campus. Herbarium voucher specimen (AshMed/01/2013/QwaHb) was prepared and deposited at the University herbarium. The root was dried in the oven at 40°C to a constant weight and pulverized using Waring Commercial Laboratory electric blender.

### Phytochemical screening

Phytochemical constituents of *M. serata* root was determined in the aqueous and ethanol extracts as well as powdered plant material adopting standard methods as described by [16-19].

### Alkaloids

Half gram of the powdered root material was stirred in 5 ml of 1% aqueous hydrochloric acid on a water bath and filtered. I ml of the filtrate was treated with few drops of mayer’s reagent and a second portion was treated same way only with Drangendorff’s reagent. Turbidity of precipitation with either of those reagents was taken as preliminary evidence for the presence of alkaloids in the extract.

### Tannins

Half gram of dried powdered sample was boiled in 20 ml of water in a test tube and filtered. Few drops of 0.1% ferric chloride was added and observed for brownish green or a blue black colouration as indication of tannins.

### Phlobatanins

Half gram of dried powdered plant material was extracted in 10 ml of distilled water. 4 ml of the filtered aqueous extract was boiled with 1% aqueous hydrochloric acid and observed for deposition of red precipitate as indication of phlobatannins.

### Saponins

Approximately 2 g of powdered material was boiled in 20 ml of distilled water in a water bath and filtered. 10 ml of the filtrate was mixed with 5 ml of distilled water and shaken vigorously and observed for a stable persistent froth. The frothing was mixed with 3 drops of olive oil and shaken vigorously again and then observed for the formation of emulsion as indication of saponin.

### Flavonoids

One gram of the powdered material was heated with 10 ml of ethyl acetate over a steam bath for 3 min. The mixture was filtered and 4 ml of the filtrate was shaken with 1 ml of dilute ammonia solution. Development of yellow colouration was taken as indication of flavonoids.

### Steroids

Two milliliter of acetic anhydride was added to 0.5 g of ethanolic extract with 2 ml H_2_SO_4._ The colour change from violet to blue or green is indication of steroids.

### Terpenoids (Salkowski test)

Five milliliter of extract was mixed with 2 ml chloroform and 3 ml H_2_SO_4_ was carefully added to form a layer. A reddish brown colouration of the interface was indication of terpenoids.

### Cardiac glycosides (Keller-Killani test)

Five milliliter of the extract was treated with 2 ml of glacial acetic acid containing one drop of ferric chloride solution. This was underplayed with 1 ml of concentrated H_2_SO_4_. A brown ring of the interface indicates a deoxysugar characteristic of cardenolides. A violet ring may appear below the brown ring, while in the acetic acid layer, a greenish ring may form just gradually throughout thin layer.

### Choice of solvents

The choices of extracting (acetone, water and ethanol) solvents were based on 1. That acetone is capable of extracting antimicrobial compounds from plant materials [20], 2. Water is the conventional solvent used in traditional medicine practice all over the world and 3. Ethanol/alcohol is the alternative solvent used in many regions to extract active ingredients from plant materials for home use.

### Preparation of extracts

20 g each of the powdered root material was extract in acetone, methanol, ethanol and distilled water respectively and placed on Labcon Platform shaker (Laboratory Consumables, PTY, Durban, South Africa) for 24 h. All extracts were filtered using number 1 Watman filter paper. The filtrates from acetone, methanol and ethanol were concentrated under reduced pressure 40°C using rotary evaporator (Cole Parmer SB 1100, Shangai, China). Acetone and ethanol used were of high analytical grade (Merck Chemicals (PTY), Wadeville, South Africa). The water extract was freeze dried using Virtis BenchTop (SP Scientific Series, USA) freeze dryer. The yields were 0.08, 1.23, 0.32 and 2.51 g for acetone, methanol, ethanol and water respectively. The individual extract was reconstituted in their respective solvent to give a stock solution of 50 mg/ml [21].

### Test organisms

Six (6) Gram-positive bacteria namely; *Staphylococcus aereus* (ATCC6538,) *Staphylococcus aereus* (OK2a), *Staphylococcus aereus* (OK2b), *Bacillus pumilis* (ATCC14884), *Streptococcus faecalis*, *Listeria* spp and twelve (12) Gram-negative bacteria, *Escherichia coli* (ATCC8739), *Shigella flexneri* (KZN)*, Shigella sonnei* (ATCC29930), *Proteus vulgaris* (CSIR0030), *Proteus vulgaris* (ATCC 6830), *Enterobacter faecalis* (KZN), *Klebsiella pneumoniae* (ATCC 13047), *K. pneumoniae, Salmonella typhi, Pseudomonas aeruginosa, Plesiomonas shigelloides* and *Acinetobacter calcaoceuticus anitratus* (CSIR) composing of reference, clinical and laboratory isolates were obtained from the Department of Biochemistry and Microbiology, University of Fort Hare, South Africa. The organisms were maintained on nutrient agar plates at 4°C in the refrigerator and were revived for bioassay by sub culturing in fresh nutrient broth (Oxoid Ltd, Basingstoke, Hampshire, England) for 24 h before being used.

### 96 wells antibacterial activity assay

The minimum inhibitory concentration (MIC) values of the extracts on each organism were determined using microplate dilution method [20], with slight modifications. Briefly, bacterial strains were cultured overnight (24 h) in autoclaved nutrient broth (Oxoid Ltd, Basingstoke, Hampshire, England), and was adjusted to a final density of 106 cfu/ml. This was used to inoculate 96-well microtitre plates containing serial two fold dilutions of the extracts (12.5 - 0.09 mg/mL) under aseptic condition. The plates were incubated under aerobic conditions at 37°C and examined after 24 h. As an indicator of bacterial growth, 40 μL of 0.2 mg/mL p-iodonitrotetrazolium (97% purity, Sigma, South Africa) solution was added to each well and incubated for 30 min at 37°C. The colourless tetrazolium salt was reduced to a red-coloured product by the biological activity of the organisms. Each treatment was performed in triplicate and complete suppression of growth at a specific concentration of oil was required for it to be declared active [20]. Sample free solutions were used as blank controls.

### Antifunfal activity assay

Anticandidal activity of *M. serata* was investigated using four non-filamentous viz: *Candida rugosa, Candida neoformans, Candida albicans* and *Trichophyton mucoides* fungal species. Fungal strains were cultured overnight (24 h) in autoclaved nutrient broth (Oxoid Ltd, Basingstoke, Hampshire, England), and was adjusted to a final density of 106 cfu/ml. This was used to inoculate 96-well microtitre plates containing serial two fold dilutions of the extracts (12.5 - 0.09 mg/mL) under aseptic condition. The plates were incubated under aerobic conditions at 25°C and examined after 24 h. As an indicator of fungal growth, 40 μL of 0.2 mg/mL p-iodonitrotetrazolium (97% purity, Sigma, South Africa) solution was added to each well and incubated for 30 min at 25°C. The colourless tetrazolium salt was reduced to a red-coloured product by the biological activity of the organisms. Each treatment was performed in triplicate and complete suppression of growth at a specific concentration of extract was required for it to be declared active [20].

### Brine shrimps lethality assay

The brine shrimps lethality test using the larvae of brine shrimp naupli, *Artemia salina* L. was carried out using the method described by [3], with slight modification. Briefly; 1 mgmL^-1^ concentration of the extract was prepared from the original stock solution of 50 mgmL^-1^ that was used for antibacterial and antifungal assays. The extract was further diluted into final concentrations of 20, 40, 60, 80 and 100 μgmL^-1^ in different vials using normal saline. Ten naupli were transferred into each vial using Pasteur pipettes and were not given food because hatched brine shrimp can survive up to 48 h without food [22], as they still feed on their yolk during this period [15]. The controls vials contain only normal saline solution and all experiments were replicated three times. After 24 h of incubation, the content of each vial was transferred into 65 mm Petri dish and examined, the number of surviving larvae counted and the percentage of death calculated. Larvae were considered dead if they did not exhibit any form of movement during several seconds of observation. Extracts are regaded as non-toxic if its LC_50_ is greater 100 μgmL^-1^ in brine shrimp lethality assay [23]. The mortality percentage and LC_50_ (lethal concentration for 50% of the population) were determined using statistical analysis and the graph of Logarithm of concentration against percentage lethality [24].

## Results

The qualitative phytochemical investigation of *M. serata* root extracts revealed the presence of tannins, phlobatanins, saponins, flavonoids, terpenoids and steroids but alkaloids and cardiac glycosides were not detected (Table 1).

**Table 1:** **Preliminary phytochemical screening of Monamotsho ( *M. serata *) root extracts**

**S/no**	**Compound**	**Present/absent**
1	Alkaloid	-
2	Tannins	+
3	Phlobatanins	+
4	Saponin	+
5	Flavonoids	+
6	Steroids	+
7	Terpenoids	+
8	Cardiac glycosides	-

+ = present, - = absent.

The minimum inhibitory concentrations (MIC) of *M. serata* root extracts against human pathogenic bacteria are presented in Table 2. The water extract inhibited all the microorganisms tested at MIC ranging from 0.78 to 6.25 mgmL^-1^, except *Eschrichia coli* ATCC 8739, *Shigella flexneri* KZN and *Staphylococcus aureus* OK2b that were inhibited at 12.50 mgmL^-1^. The ethanol extract also inhibited both Gram-negative and Gram-positive bacterial strains at MIC range of 0.39 - 1.56 mgmL^-1^, whereas the methanol extract did the same by inhibiting all organisms tested at 0.39 to 3.13 mgmL^-1^. The acetone extract suppressed the growth of all tested organisms at MIC range of 0.09 -0.78 mgmL^-1^ except *Shigella flexneri* KZN that was inhibited at 1.56 mgmL^-1^.

**Table 2:** **Minimum inhibitory concentrations (MIC) of Monamotsho (*M. serata*) root extracts against human pathogenic bacteria**

**Organism**	**Gram+/−**	**Extract (mgmL**^**-1**^**)**			
		**Water**	**Ethanol**	**Methanol**	**Acetone**
*Acetenobacter Calcoaceticus anitratus* CSIR	-	6.25	0.78	1.56	0.39
*Aeromonas hydrophila*	-	3.13	0.39	0.78	0.09
*Bacillus pumilis* ATCC 14884	+	6.25	0.78	1.56	0.39
*Enterobacter faecalis* KZN	-	3.13	0.78	1.56	0.78
*Escherichia coli* ATCC	-	12.50	0.78	3.13	0.78
*Klebsiella pneumoniae*	-	6.25	0.78	3.13	0.78
*Klebsiella pneumoniae* ATCC 13047	-	1.56	0.39	1.56	0.19
*Listeria* spp	+	1.56	0.39	1.56	0.09
*Plesiomonas shigelloides*	-	3.13	0.39	1.56	0.39
*Proteus vulgaris* CSIR 0030	-	3.13	0.78	3.13	0.78
*Proteus. vulgaris* ATCC 6830	-	3.13	0.19	0.39	0.09
*Pseudomonas aeruginosa*	-	1.56	0.39	1.56	0.09
*Salmonella typhi*	-	6.25	0.39	1.56	0.09
*Salmonella typhimurium*	-	0.78	0.39	0.78	0.09
*Shigella flexneri* KZN	-	12.50	0.78	3.13	1.56
*Shigella sonnei* ATCC 29930	-	6.25	0.78	1.56	0.78
*Staphylococcus aureus* ATCC 6538	+	6.25	0.78	3.13	0.78
*Staphylococcus aureus* OK2a	+	6.25	0.78	1.56	0.09
*Staphylococcus aureus* OK2b	+	12.50	1.56	3.13	0.78
*Streptococcus faecalis*	+	3.13	0.39	1.56	0.09

The minimum inhibitory concentrations (MIC) of *M. serrata* root extracts against Candida species and *Trichophyton mucoides* are presented in Table 3. The acetone extract was able to inhibit the growth of all Candida species and *Trichophyton mucoides* at MIC range of 0.13 to 0.39 mgmL^-1^, whereas, the ethanol, methanol and water extracts suppressed the growth of all fungi tested at 0.78, 1.56 and 3.13 mgmL^-1^ respectively.

**Table 3:** **Anticandida activity of Monamotsho (*M. serrata*) root extracts against Candida species and *Trychophyton mucoides***

**Extract (mg/mL**^**-1**^**)**					
**S/no**	**Organism**	**Acetone**	**Ethanol**	**Methanol**	**Water**
1	*Candida rugosa*	0.39	0.78	1.56	3.13
2	*Candida neoformans*	0.19	0.78	1.56	3.13
3	*Candida albicans*	0.13	0.78	1.56	3.13
4	*Trichophyton mucoides*	0.39	0.78	1.56	3.13

The effects of *M. serata* root extracts on the rate of mortality of brine shrimps larvae are presented in Table 4. The methanol and ethanol root extracts of *M. serata* displayed potent cytotoxic potential against *Artemia salina* (Brine shrimps larvae) in that the LC_50_ of the two extracts were 0.26 and 0.18 μg/mL respectively

**Table 4:** **Cytotoxic effect of water and ethanol roots extracts of Monamotsho (*M. serata*) on brine shrimps (*Artemia salina*) larvae (% mortality)**

**Conc. (μg/mL)**	**Total no.**	**Number of dead (24 hr)**				**% mortality**	
		**Control**	**Methanol**	**Ethanol**	**Log of conc.**	**Methanol**	**Ethanol**
	10	0			-	0	0
20	10	0	2	2	1.30	20	20
40	10	0	4	2	1.60	10	10
60	10	0	1	1	1.77	10	10
80	10	0	5	5	1.90	50	50
100	10	0	10	10	2.00	100	100
LC_50_			0.26 μg/mL	0.18 μg/mL			

Generally, all the extracts including water displayed moderate to strong antibacterial and anticandida activity but the best results were obtained from the acetone extract that inhibited most of the bacterial strains at MIC less than 0.78 mgmL^-1^.

## Discussion

The antimicrobial screening of traditional medicinal plants has been the source of innumerable therapeutic agents [25,26]. While many complementary and alternative medicines have enjoyed increasing popularity recently, efforts made to validate their use have seen their assumed effective therapeutic properties being increasingly scrutinized *in vitro* and *in vivo*. The reason being that large numbers of antimicrobial agents derived from medicinal plants are readily available for treating various diseases caused by microorganisms [25]. Recent reports have shown that there is reduction in the discovery of new antimicrobials agents globally [26], coupled with alarming cases of drugs resistant to available antimicrobials. Minimum inhibitory concentration (MIC) is used as an index for measuring the efficacy of antibacterial agents [27].

The results from the present study has shown that all the extracts including water can inhibit the growth of *Acinetobacter calcaoceticus anitratus* at MIC range of 0.39 -6.25 mgmL^-1^. This organism is ubiquitous in nature and found as normal flora of the skin and throat of human beings along with other saprophytes. However, it is proved beyond doubt that this organism is opportunistic in nature with highly variable degree of virulence causing meningitis, fulminating septicaemia, pulmonary and ophthalmic infections, chronic sinovitis, skin diseases and wound infections. Although the organism appear to have little invasive power and depend upon a pre-existing break in the normal body defense like a surgical wound to cause disease, they may also cause infections in debilitated patients or those administered immunosuppressive drugs [28]. The ability of *M. serata* root extracts to inhibit the growth of this organism is an indication that the plant can be employed in the management of array of diseases caused by Acinetobacter species.

*Aeromonas hydrophila* also are ubiquitous microorganisms found in both aquatic and environmental habitats such as estuary, sediment, sea water, sea grass, sea weed, waste and used water, food and drinking water [29-32]. Among the species in the genus Aeromonas, *A. hydrophila* is the most studied due to its presence in food and water [33,34], and its antibiotic resistance and ability to cause infections in humans and livestock [35]. *A. hydrophila* has been identified as causative agent of human diseases such as diarrhea, septicaemia, meningitis, wound infections as a result of exposure to contaminated marine environment [32,35,36]. The result from this study showed that all the extracts from the root of *M. serata* inhibited *A. hydrophila* at very low minimum inhibitory concentrations. This is indicative of the fact that *M. serata* extracts in any form (infusion, decoction or maceration) can be used to treat diseases related to *A. hydrophila* infections mostly in areas with poor water management systems.

Shigella species are known to cause shigellosis, an important cause of worldwide morbidity and mortality among young children and old people for which treatment with antimicrobial agents is limited [25]. The results from the present study showed that *M. serata* ethanol root extract inhibited both strains of Shigella at 0.78 mgmL^-1^. We earlier reported that Moxypen (Penicillin) suppressed the growth of a strain of *Shigella flexneri* KZN at 1.56 mgmL^-1^[37]. The outcome of this study clearly indicated that *M. serata* root extracts can be good replacement for Moxypen or other antibiotics in the Eastern Free State of South Africa where most of the inhabitants are low income earners who cannot afford western medications.

*Pseudomonas aeruginosa* an important Gram-negative opportunistic pathogen, is the primary cause of hot tub folliculitis, otitis externa, as well as the principal cause of morbidity and mortality in cystic fibrosis chronic pulmonary infection patients [38] and a leading cause of progressive loss of lungs function and early death [39]. *P. aeruginosa* is highly ubiquitous in water systems, and has intrinsic antimicrobial resistance due to low outer membrane permeability, as well as an extensive efflux pump system [40-42]. The organism is known to be resistant to most available antimicrobial agents but the root extract of *M. serata* was able to inhibit *P. aeruginosa* at MIC range of 0.09 to 1.56 mgmL^-1^. The present study agrees with some earlier reports that medicinal plants could be the alternative source of potent antimicrobials [25,43]. This study presents *M. serata* as likely new source of potent antibacterial and antifungal agents with significant activity against infective microorganisms.

All the root extracts of *M. serata* were able to inhibit the growth of both clinical and reference isolates of *S. aureus* at MIC ranging from 0.09 – 3.13 mgmL^-1^. Staphylococci are known to cause food poisoning resulting in nausea, vomiting, diarrhoea and dehydration. They are also the causative agents of impetigo, cellulitis, scalded skin syndrome and mastitis in breast feeding mothers. *Staphylococcus aureus*, the most virulent of the many staphylococcal species, has demonstrated its versatility by remaining a major cause of morbidity and mortality despite the availability of numerous effective antistaphylococcal antibiotics. *S. aureus* is a pluripotent pathogen; causing disease through both toxin-mediated and non–toxin-mediated mechanisms. The effectiveness of the extracts to inhibit all Staphylococci tested in this study is indicative of antistaphylococcal potential of this plant.

Candida species are known to cause candidiasis which encompasses infections that range from superficial, such as oral thrush and vaginitis, to systemic and potentially life-threatening diseases. *Candida* infections of the latter category are also referred to as candidemia and are usually confined to severely immunocompromised persons, such as cancer, transplant, and AIDS patients as well as non-trauma emergency surgery patients [44]. Superficial infections of skin and mucosal membranes by *Candida* causing local inflammation and discomfort are common in many human populations [45,46]. The capability of the extracts to suppress Cadnida strains at MIC ranging from 0.13 to 3.13 mg/mL^-1^ is encouraging as the organism is rarely susceptible to plant extracts action at low concentrations. Similarly, the extracts suppressed the growth of *Trichophyton mucoides* at MIC ranging from 0.39 – 3.13 mgmL^-1^. It has been reported that these fungi are major cause of destruction and biochemical changes of food commodities, candiasis and candida vaginatis, producing mycotoxins and carcinogens as well as serious threat to human health [25,47]. The present study has shown that extract from *M. serata* root can suppress the growth of these organisms at MIC less than 1 mgmL^-1^.

The *in vivo* lethality in a simple zoological organism, such as brine shrimp lethality test (BST) might be used as a simple tool to guide screening and fractionation of physiological active plant extracts, where one of the simplest biological responses to monitor is lethality, since there is only one criterion; either dead or alive [48,49]. In the present study, ethanol and methanol root extracts of *M. serata* displayed significant lethality against brine shrimp naupli with LC_50_ of 0.26 and 0.18 μgmL^-1^ respectively. There is a general toxicity test agreement that LC_50_ above 100 μgmL^-1^ is non-toxic while that below 100 μgmL-1 is indicative of toxicity. The significant lethality of the extracts from the root *M. serata* is an indication of the presence of potent cytotoxic compounds. The outcome of the present study presents *M. serata* root as a promising natural source of anti-tumor compounds and it deserves further explorations considering the rate of cancer spread in South Africa.

The results from this study have shown that extracts from this plant could inhibit the growth of several human pathogenic bacteria and fungi species at relatively low concentrations. This is an indication of the broad spectrum antimicrobial potential of *M. serata* that could make the species a candidate for antibiotic bioprospecting. The ability of the extracts from this herb to effectively suppress the growth of both human pathogenic bacteria and fungi at relatively low concentrations further validates the folkloric use of the species for the treatment of various human and livestock diseases. The observed antimicrobial activities in this study could be attributed to the presence of phytochemicals like saponins, tannins and phenolics [50].

## Conclusion

Results from this study have shown that *M. serata* possessed broad spectrum antimicrobial activity; it is therefore a wonderful addition to the South African list of plants for antibiotics bioprospecting. Also, the cytotoxic effect displayed by the extracts from the root of this plant also makes the plant a candidate for antitumor bioprospecting for the South African population. Further study is ongoing on the isolation, purification and structural elucidation of the bioactive compounds in this plant.

## Abbreviations

MIC: Minimum inhibitory concentrations; KZN: KwaZulu Natal; UFS: University of the Free State.

## Competing interests

The author declares that there are no competing interests.

1. I am employed by the University of the Free State, South Africa and the article-processing charge shall be paid from my research account with entity number 211427604.

2. I am employed as a lecturer and researcher in a public university. I do not belong to the private sector I therefore hold no stocks or shares in any organization.

3. No patent and no intention of patenting any of the content of the manuscript.

4. As an employee of the University of the Free State, I state categorically that my employer has not and will not apply for patent on the content of this manuscript.

5. There are no financial competing interests.

6. There are no non-financial competing interests.

7. I unequivocally declared that there is no political, religious, personal, ideological, academic, intellectual and commercial competing interest on this manuscript.

## Authors’ contributions

AOTA conceptualize, designed, conducted all experiments and drafted the manuscript.

## Authors’ information

AOTA, Phytomedicine and Phytopharmacology Research Group, Department of Plant Sciences,

University of the Free state, Private Bag X13, Phuthaditjhaba 9866, South Africa.

## Pre-publication history

The pre-publication history for this paper can be accessed here:

http://www.biomedcentral.com/1472-6882/13/163/prepub
